# Ascorbate synthesis as an alternative electron source for mitochondrial respiration: Possible implications for the plant performance

**DOI:** 10.3389/fpls.2022.987077

**Published:** 2022-11-23

**Authors:** Isabelle Faria Matos, Luis Miguel Mazorra Morales, Diederson Bortolini Santana, Gláucia Michelle Cosme Silva, Mara Menezes de Assis Gomes, Ricardo Antônio Ayub, José Hélio Costa, Jurandi Gonçalves de Oliveira

**Affiliations:** ^1^Plant Genetic Breeding Laboratory, Center for Agricultural Sciences and Technologies, Universidade Estadual do Norte Fluminense Darcy Ribeiro (UENF), Campos dos Goytacazes, RJ, Brazil; ^2^Plant Physiology Institute, National University of La Plata, La Plata, Argentina; ^3^Laboratory of Biotechnology Applied to Fruit Growing, Department of Phytotechny and Phytosanitary, Universidade Estadual de Ponta Grossa, Ponta Grossa, PR, Brazil; ^4^Functional Genomics and Bioinformatics, Department of Biochemistry and Molecular Biology, Universidade Federal do Ceará, Fortaleza, CE, Brazil; ^5^Non-Institutional Competence Focus (NICFocus) ‘Functional Cell Reprogramming and Organism Plasticity’ (FunCROP), coordinated from Foros de Vale de Figueira, Alentejo, Portugal

**Keywords:** alternative oxidase, l-galactone-1,4-lactone dehydrogenase, respiration, cytochrome c, mETC, mitochondrial alternative pathway

## Abstract

The molecule vitamin C, in the chemical form of ascorbic acid (AsA), is known to be essential for the metabolism of humans and animals. Humans do not produce AsA, so they depend on plants as a source of vitamin C for their food. The AsA synthesis pathway occurs partially in the cytosol, but the last oxidation step is physically linked to the respiratory chain of plant mitochondria. This oxidation step is catalyzed by l-galactono-1,4-lactone dehydrogenase (l-GalLDH). This enzyme is not considered a limiting step for AsA production; however, it presents a distinguishing characteristic: the l-GalLDH can introduce electrons directly into the respiratory chain through cytochrome c (Cytc) and therefore can be considered an extramitochondrial electron source that bypasses the phosphorylating Complex III. The use of Cytc as electron acceptor has been debated in terms of its need for AsA synthesis, but little has been said in relation to its impact on the functioning of the respiratory chain. This work seeks to offer a new view about the possible changes that result of the link between AsA synthesis and the mitochondrial respiration. We hypothesized that some physiological alterations related to low AsA may be not only explained by the deficiency of this molecule but also by the changes in the respiratory function. We discussed some findings showing that respiratory mutants contained changes in AsA synthesis. Besides, recent works that also indicate that the excessive electron transport *via* l-GalLDH enzyme may affect other respiratory pathways. We proposed that Cytc reduction by l-GalLDH may be part of an alternative respiratory pathway that is active during AsA synthesis. Also, it is proposed that possible links of this pathway with other pathways of alternative electron transport in plant mitochondria may exist. The review suggests potential implications of this relationship, particularly for situations of stress. We hypothesized that this pathway of alternative electron input would serve as a strategy for adaptation of plant respiration to changing conditions.

## Introduction

**Figure 1:**
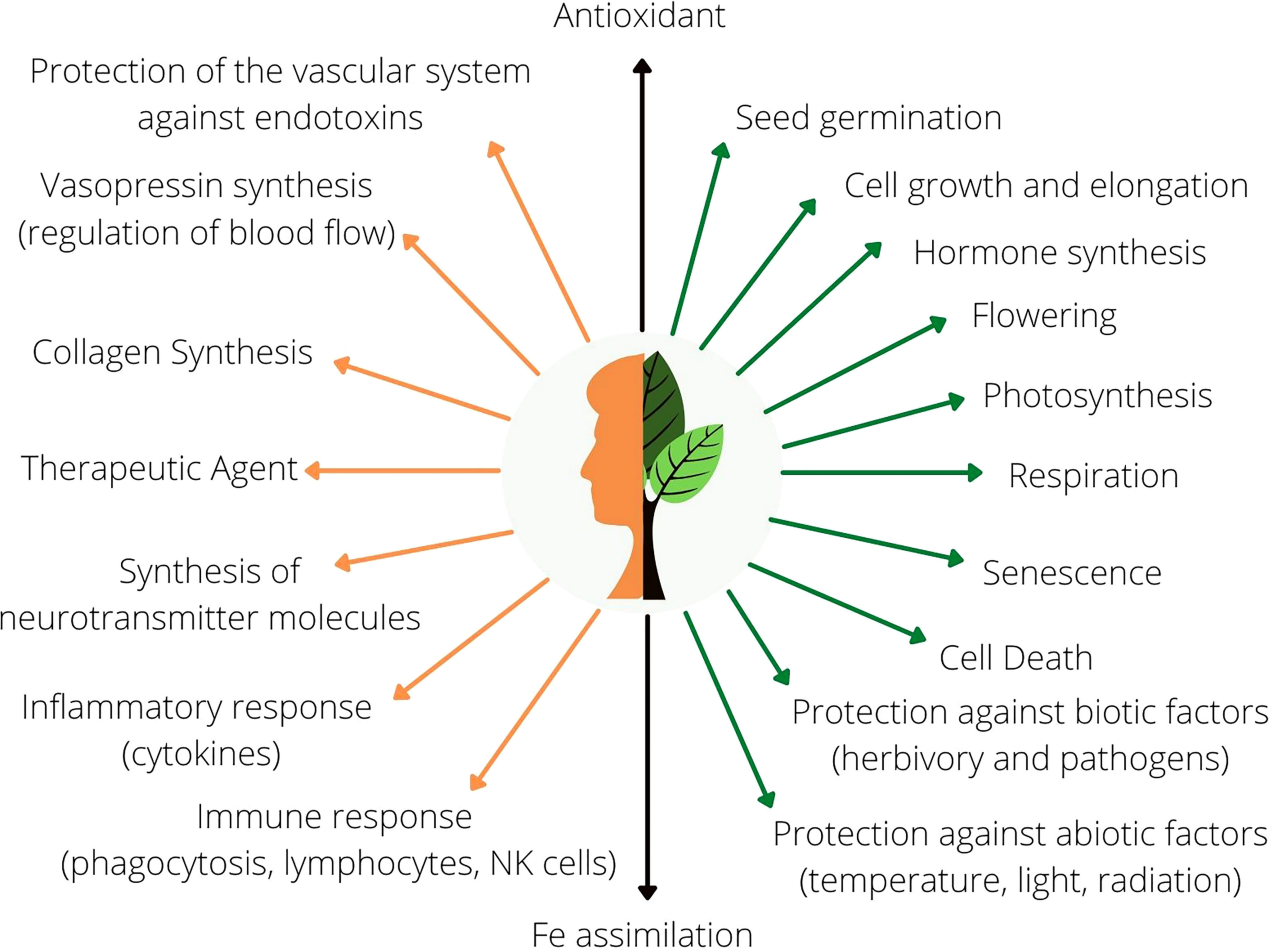
General overview of the functions of AsA in animal (orange) and plant (green) metabolism. Black arrows represent common molecular functions in both human and plant metabolism.

Ascorbic acid (AsA), commonly called vitamin C, is an important antioxidant essential to animal and plant metabolism (**Figure 1**). The pathways of AsA synthesis have not been fully elucidated in plants. Indeed, four possible pathways have been proposed, with the so-called “Smirnoff-Wheeler” or “d-mannose/l-galactose” pathway being the best characterized. In plant cells, AsA synthesis includes the conversion of sugars related to the metabolism of cell walls (d-mannose and l-galactose) into the immediate AsA precursors, l-galactono-1,4-lactone (l-GalL) and l-gulono-1,4-lactone (l-GulL) through reactions of isomerization, phosphorylation, epimerization, and oxidations (Smirnoff, 2001; Smirnoff, 2018). In the past, most studies have focused on elucidating the key enzymes that catalyze these reactions as well as the regulation and the physiological roles of AsA synthesis (Horemans et al., 2000; Imai et al., 2009; Hemavathi et al., 2010; Cruz-Rus et al., 2011; Zhou et al., 2012; Gallie, 2013; Castro et al., 2015). There is considerable consensus in the literature about the regulation of key biosynthetic enzymes by light, the roles of this molecule as an antioxidant or as an enzymatic co-factor and its implication in essential processes of plant growth and development, particularly under stress conditions (Gatzek et al., 2002; Mastropasqua et al., 2012; Wheeler et al., 2015; Ntagkas et al., 2018; Bulley et al., 2021).

The concentrations of AsA in different cellular compartments can be quite variable (Palma et al., 2006; Luschin-Ebengreuth and Zechamann, 2016). AsA is not synthesized by chloroplast, but AsA mostly accumulates in this organelle. It is evident that this molecule is implicated in events related to light capture and electron transport by photosynthetic pigments, the AsA-GSH cycle in chloroplast, and other processes (Yabuta et al., 2007; Nunes-Nesi et al., 2008; Zheng et al., 2021). On the other hand, there are data showing that AsA accumulates in mitochondria at lower concentrations than in chloroplasts (Zechmann et al., 2011). However, the last step of AsA synthesis is linked with a mitochondrial-localized enzyme called l-GalL dehydrogenase (l-GalLDH, EC 1.3.2.3) (Bartoli et al., 2000). This enzyme catalyzes the last oxidation reaction during AsA synthesis and is strongly induced by high light (Bartoli et al., 2006). Humans do not produce AsA because l-GulL oxidase (l-GulLO) was lost during evolution (Wheeler et al., 2015). This enzyme is not considered to be a limitation for AsA production (Gatzek et al., 2002; Fenech et al., 2021). Thus, l-GalLDH has received relatively less attention as compared to other key biosynthetic enzymes. However, an aspect of the action of l-GalLDH could have unexpected implications for the function of plant mitochondria and consequently overall plant physiology: This enzyme is able to introduce electrons into the mitochondrial respiratory chain using cytochrome c (Cytc) as a direct electron acceptor (Leferink et al., 2008). The plant mitochondria are also unique in that they can receive electrons through alternative pathways of NAD(P)H oxidation (Rasmusson et al., 2020). Thus, plant mitochondria can partition the electron flux towards the alternative pathways, which is particularly evident under stressful conditions. The mitochondrial alternative pathway is the most light-responsive component of the mitochondrial respiratory chain (Vishwakarma et al., 2014; Jiang et al., 2019). It is thought that these pathways of alternative electron entry can be connected to the electron movement through ubiquinone (UQ) and the alternative oxidase (AOX). However, there has been little debate in the literature regarding a possible link of electron transport with the l-GalLDH, especially the implications of the l-GalLDH’s ability to introduce electrons through Cytc and not through UQ. Observations from studies with respiratory mutants and l-GalLDH deficient plants have shown how the amount of AsA changes when the electron flux is altered through mitochondrial respiration (Vidal et al., 2007; Meyer et al., 2009). In addition, it is not yet well-understood why increases in the alternative respiration enhance AsA under light (Bartoli et al., 2006) or if this positive relationship may also occur under conditions in which AsA synthesis is decreased. This review is an attempt to offer a new perspective to explain the possible inter-dependency between AsA synthesis and mitochondrial respiration. To this end, the review hypothesizes that the alternative introduction of electrons into the respiratory chain *via* l-GalLDH can influence the function of the mitochondrial electron transport.

## Overview of the pathways of AsA synthesis

**Figure 2:**
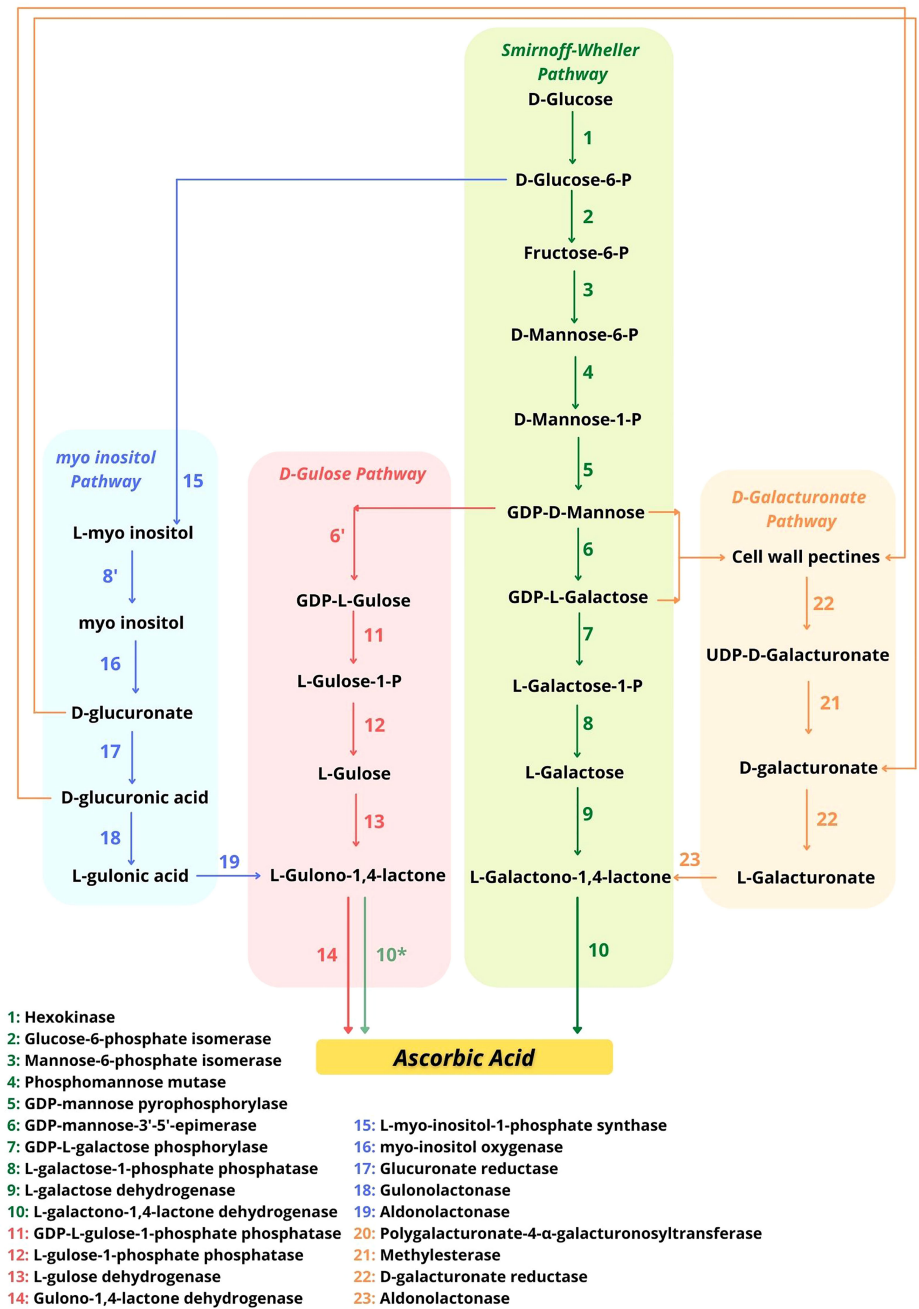
Possible pathways for AsA synthesis in plants indicating the potential interaction points. Adapted from Smirnoff (2018). The possibility that l-GalLDH uses l-GulL as a substrate to form AsA (Leferink et al., 2008) is indicated with an asterisk (*) in the d-Gulose pathway.

In addition to the previously mentioned Smirnoff-Wheeler pathway, three other pathways for AsA synthesis have been described (**Figure 2**).The Smirnoff-Wheeler is the main pathway of AsA production plants, being the best described pathway, consisting of 10 steps until AsA synthesis from the glucose molecule (**Figure 2**). However, as the two initial steps utilize substrates from the cellular hexose phosphate pool and, therefore, are not exclusive to the AsA synthesis pathway, the Smirnoff-Wheeler pathway properly initiates from the activity of mannose-6-phosphate isomerase. The first nine steps of this pathway occur in the cytosol and culminate in the formation of the precursor l-GalL, which is converted to AsA by l-GalLDH within the mitochondria (Wheeler et al., 1998). For some authors, l-GalLDH in plants (and in Euglena) is highly specific for l-GalL (Ôba et al., 1995; Yabuta et al., 2000; Smirnoff, 2001; Leferink et al., 2008), not using l-GulL (Wheeler et al., 2015) or using l-GulL at a low rate in bean shoots, strawberry fruit, and potato tuber tissues (Baig et al., 1970; Ôba et al., 1995) (**Figure 2**).

**Figure 3:**
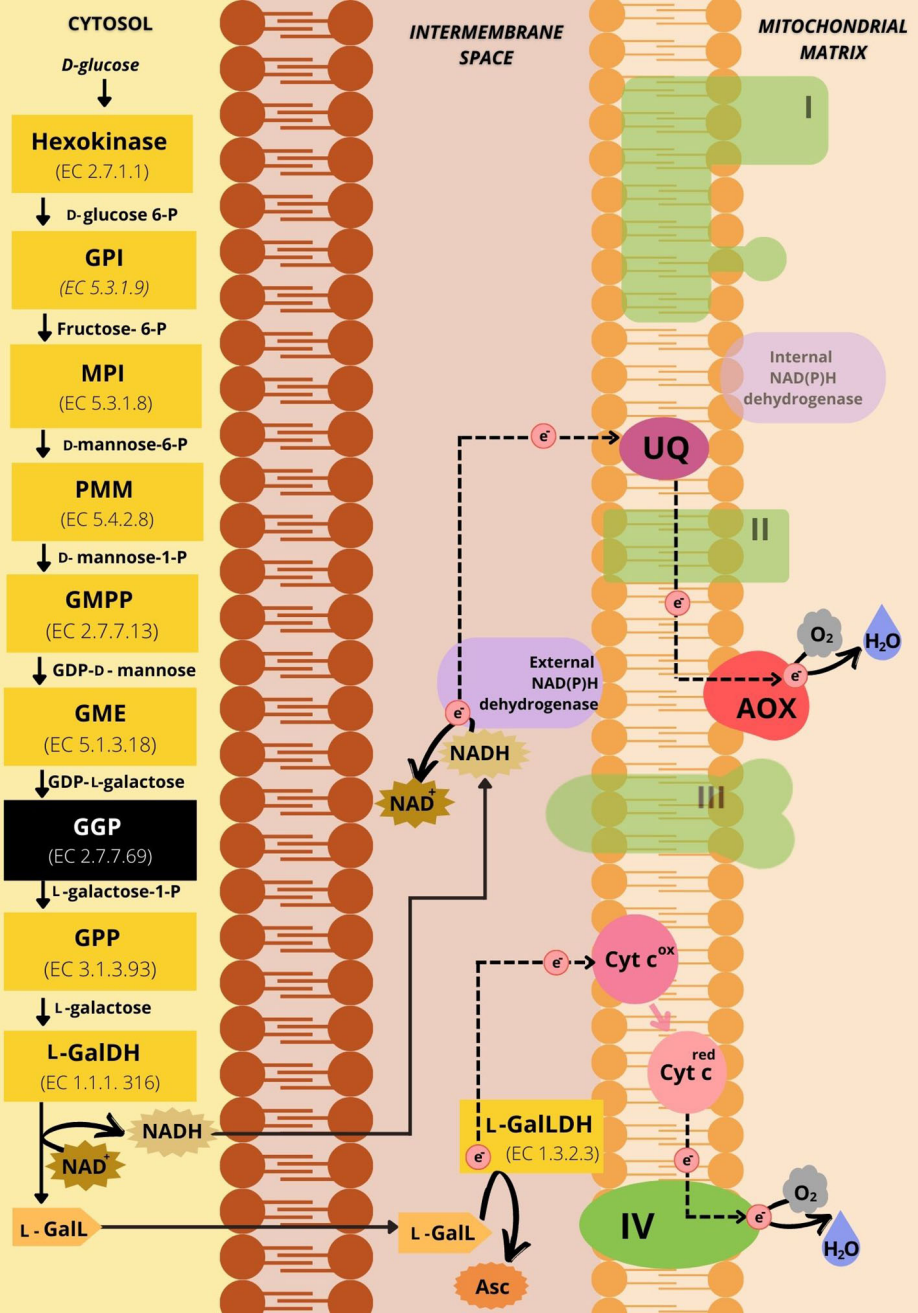
Representative scheme of the d-mannose/l-galactose pathway of ascorbic acid synthesis and its connections with the mETC. The yellow boxes represent the enzymes involved in the 10 steps of the pathway. The black box has the GDP-l-galactose phosphorylase, the enzyme that catalyzes the limiting step for AsA synthesis. The substrates of the enzymatic reactions are described between the boxes. The dotted black arrows represent the path of electrons in the mETC. In green are the respiratory complexes indicated by Roman numerals corresponding to the I-IV Complexes. The shaded green is indicative of respiratory protein components not directly engaged with electron flux during the synthesis of ascorbic acid in light. In purple are the alternative NAD(P)H dehydrogenases, highlighting the external NAD(P)H dehydrogenase as the location of the potential regeneration of NAD^+^. The alternative oxidase is in red and Cytc is in pink. The ubiquinone pool is represented in magenta. NAD^+^ and NADH are represented by spiked outlines. The substrate l-galactone-1,4-lactone (l-GalL) (in light orange) is oxidized by l-GalLDH using Cytc as the electron acceptor to produce ascorbate (Asc) (in dark orange). GPI: Glucose-6-phosphate isomerase. MPI: Mannose-6-phosphate isomerase. PMM: Phosphomannose mutase. GMPP, GDP-mannose pyrophosphorylase; GME, GDP-mannose-3’-5’-epimerase; GGP, GDP-l-galactose phosphorylase; GPP, l-galactose-1-phosphate phosphatase; l-GalDH, l-galactose dehydrogenase; l-GalLDH, l-galactono-1,4-lactone dehydrogenase; l-GalL, l-Galactono-1,4-lactone; Asc, Ascorbate; NAD^+^, Nicotinamide adenine dinucleotide oxidized; NADH, Nicotinamide adenine dinucleotide reduced; Cyt c^ox^, Cytochrome c in oxidated state; Cyt c^red^, Reduced cytochrome c; AOX, Alternative oxidase; UQ, ubiquinone; I, NADH-ubiquinone oxidoreductase; II, succinate-ubiquinone oxidoreductase; III, ubiquinol-cytochrome c oxidoreductase; IV, cytochrome c oxidase; H^+^, proton; e^-^, electron.

As l-GalLDH is mitochondrial and introduces electrons directly to the Cytc (**Figure 3**), the possible implications of this link will be of interest to this review. In addition, l-galactone-1,4-lactone is synthesized from the oxidation of l-galactose (l-Gal) by the NAD-dependent l-galactose dehydrogenase (l-GalDH). The enzyme l-GalDH generates cytosolic NADH, which has to be re-oxidized to allow the availability of NAD^+^ and therefore sustain the reaction (**Figure 3**). Potentially, the regeneration of NAD^+^ may be accomplished through external mitochondrial NADH dehydrogenases (NADH DHs) (**Figure 3**). l-Galactose is generated from d-mannose-1-phosphate by the conversion of guanosine diphosphate (GDP)-d-mannose to GDP-l-galactose by GDP-mannose-3’,5’-epimerase which is then converted to l-Gal. d-Mannose-1-phosphate is synthesized from mannose-6-phosphate which is formed by mannose-6-phosphate isomerase from fructose-6-phosphate. Fructose-6-phosphate is the result of the action of glucose-6-phosphate isomerase on d-glucose-6-phosphate, generated from d-glucose by the action of hexokinase. According to Bulley et al. (2009) GDP-l-galactose phosphorylase catalyzes the main control point of AsA biosynthesis through the Smirnoff-Wheeler pathway in plants (**Figure 3**).

As shown in **Figure 2**, the “d-galacturonate” pathway uses products from the degradation of cell wall pectins and also leads to the formation of the same precursor of the d-mannose/l-galactose pathway, l-GalL (Agius et al., 2003). The other two possible pathways of AsA synthesis culminate in the formation of the precursor l-gulono-1,4-lactone (l-GulL) instead of l-GalL. l-GulL is utilized by l-GulL-1,4-lactone oxidase (l-GulLO: l-GulL oxidase or l-GulL dehydrogenase, EC 1.1.3.8). It is attributed to the location of l-GulLO in the lumen of the endoplasmic reticulum (ER) (Wheeler et al., 2015), but evidence of this location in the cellular environment is lacking. Between these two pathways, the “d-Gulose” pathway uses GDP-l-Gulose formed from GDP-d-mannose (**Figure 2**), whose formation follows the same route as described in the d-mannose/l-galactose pathway for AsA production (Jain and Nessler, 2000). Finally, the last known possible pathway is called the “myo-inositol pathway” (**Figure 2**), where glucose is used for the production of myo-inositol, which through other steps will be converted into the precursor l-GulL (Lorence et al., 2004). However, some questions about this route are still open (Kavkova et al., 2019).

### Proteins regulating AsA synthesis

Plant AsA biosynthesis regulation is multifaceted, occurring at many levels and in response to several stimuli (Conklin et al., 2013). This Section will only offer an overview about this aspect of AsA synthesis as it has been reviewed previously (Foyer et al., 2020; Rosado-Souza et al., 2020; Viviani et al., 2021). Several proteins that directly regulate AsA synthesis at both transcriptional and post-transcriptional level have been discovered. It includes AMR1 (ascorbic acid mannose pathway regulator 1), which transcriptionally represses GDP-l-galactose phosphorylase (GGP) *VTC2* (Zhang et al., 2009). AMR1 also inhibits AsA synthesis during leaf aging and in response to ozone (Zhang et al., 2009). Overexpression of *AMR1* in Arabidopsis causes a decrease in AsA levels and sensitivity to ozone, while the *amr1* mutant contained higher AsA levels and ozone tolerance than the wild-type (Zhang et al., 2009). The regulatory factor, ABI4, also transcriptionally represses *VTC2* (Zhang et al., 2009). It was further demonstrated that ABI4 is required for the regulation of growth and jasmonate-dependent defense signalling pathways by AsA (Kerchev et al., 2011). It is known that ABI4 is a component mediating ABA signaling and it also regulates the mitochondrial retrograde response in plants (Giraud et al., 2009). In addition, the PTPN protein, encodes an enzyme with nucleotidase activity. It is required to regulate AsA biosynthesis *via VTC2* (Zhang et al., 2020). Another regulator is AtERF98, which enhances expression of AsA synthesis genes in the d-mannose/l-galactose (d-Man/l-Gal) AsA pathway and salt resistance (Zhang et al., 2012). At post-transcriptional level, the photomorphogenic factor COP9 signalosome subunit 5B (*CSN5B*) can interact with the VTC1 protein and promote degradation of AsA biosynthetic gene (*VTC1*) *via* the 26S proteasome pathway when plants are grown in the dark (Wang et al., 2013).

## The alternative electron entry into plant mETC

**Figure 4:**
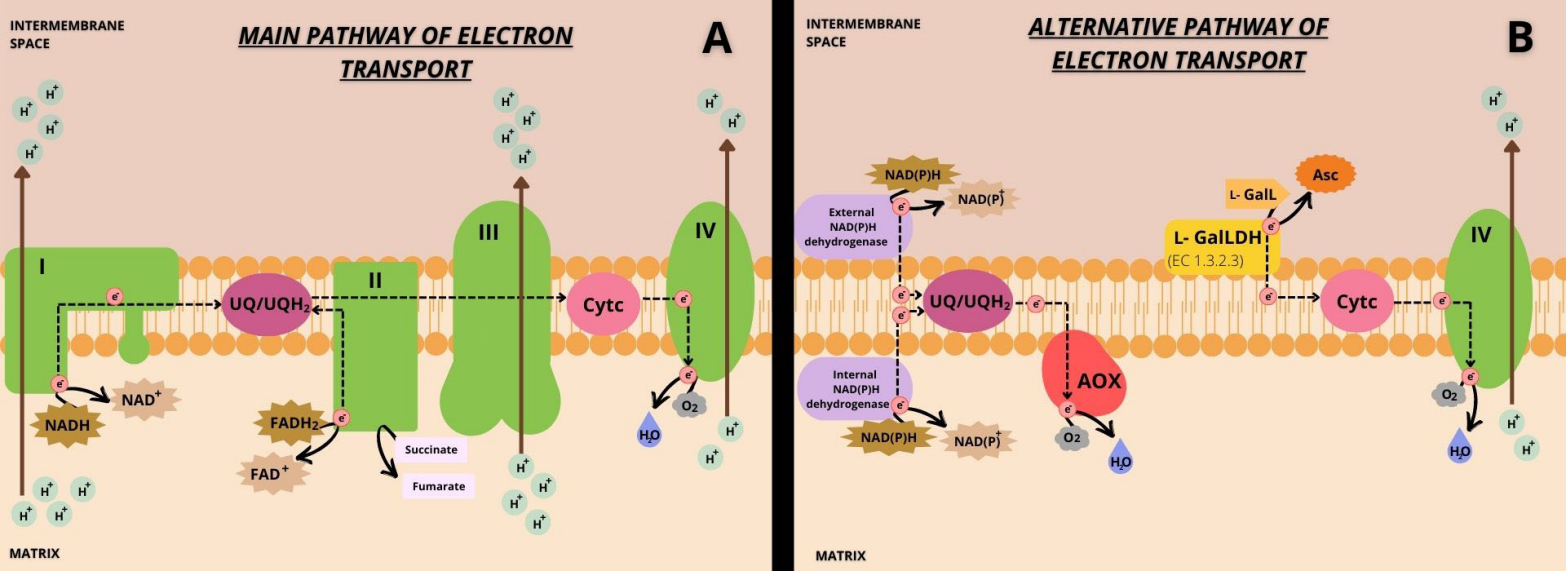
Illustrative scheme of the main **(A)** and alternative **(B)** pathways of Cytc reduction with the transport of electrons and proton pumping in mETC. In the main pathway **(A)**, electrons enter the mETC through Complexes I and II and may be used for UQ reduction. This is commonly referred to as the main pathway of UQ reduction by Complexes I and II. Then, UQH_2_ can reduce Cytc, passing electrons through Complex III. Cytc is re-oxidized transferring electrons to Complex IV, which can reduce molecular oxygen. In the alternative pathway **(B)**, electrons enter the mETC through alternative NAD(P)H dehydrogenase [NAD(P)H DH] and through l-GalLDH. Electrons entering through alternative NAD(P)H DH are mostly transferred to AOX *via* UQ/UQH_2_, reducing molecular oxygen. This is called the alternative pathway of UQ reduction by alternative NAD(P)H dehydrogenases. Electrons entering through l-GalLDH are not engaged with UQ/UQH_2_ and are directly transferred to Cytc, and this is thus called the alternative pathway of Cytc reduction by l-GalLDH. Finally, Cytc would be re-oxidized by Complex IV coupled with reduction of the molecular oxygen. The black dotted arrows indicate the electron transport pathways within the mETC. The brown arrows represent the site of proton transport that occurs from the matrix to the mitochondrial intermembrane space. The transmembrane location of the l-GalLDH enzyme is indicated by the yellow folded shape, and the enzyme activity shows the association of the enzyme with electron flow in the mETC. In green are the four mETC Complexes indicated by the corresponding Roman numerals, I-IV. AOX is represented in red and the internal and external NADP(H) dehydrogenases in purple. The ubiquinone pool and cytochrome c are represented in magenta and pink, respectively.

Although the mETC of plants shares similar features with the mETC of animal mitochondria, there are critical differences in composition and functions (Møller et al., 2021). The classical view is that electrons from the matrix NADH oxidation in Complex I are transferred to the UQ, reducing it to UQH_2_, while protons are transported to the inter-membrane space (IMS) (Møller, 2001; Ghifari and Murcha, 2020) (**Figure 4A**). Electrons from the oxidation of succinate to fumarate, which occurs through FADH_2_ by succinate dehydrogenase, or simply by the mETC Complex II are also transferred *via* UQ (**Figure 4A**). Although, beyond the classical Complexes I and II found in animal mitochondria, plant mitochondria present alternative pathways for the entry of electrons. Three alternative NAD(P)H dehydrogenase [NAD(P)H DH] are facing the matrix (NADH DHs: NDA1-2 and NADPH DH: NDC1) and four others the intermembrane space (NADH DHs: NDB2-4 and NADPH DHs: NDB1) (Rasmusson et al., 2020). Thus, mETC in plants has alternative external and internal NADH DH (**Figure 4B**) that can directly use the matrix or cytosolic NAD(P)H and therefore they can be an alternative electron source. It is believed that these alternative matrix or cytosolic NAD(P)H DH are competing with the activity of Complex I, and some are activated even when Complex I is inhibited (Gazizova et al., 2020). The role of alternative NADH DH is not fully defined; however, they may be linked with plant responses to stress. A major external NADH DH, Arabidopsis NDB2, is linked with plants more tolerant to drought and high light (Sweetman et al., 2019).

On the other hand, plant mETC also contains an electron-transfer flavoprotein/electron-transfer flavoprotein:ubiquinone oxidoreductase (ETF/ETFQO) Complex, which can directly entry electrons into mETC through UQ (Araújo et al., 2010). As it can be noted, all these enzymes related to alternative electron input pathways have in common that transfer electrons to the ubiquinone (UQ). In this review, the reduction of UQ *via* these matrix and cytosolic NAD(P)H DH will be called the “alternative UQ reduction” (**Figure 4B**) to differentiate it from the main UQ reduction by electrons from Complexes I and II (**Figure 4A**).

The origin of NAD(P)H as substrates for these alternative dehydrogenases is diverse. The mitochondrial matrix NADH can come from the TCA cycle or the photorespiratory process. NADPH can be generated by the isocitrate dehydrogenase, mainly in photosynthetic tissues (Igamberdiev and Gardeström, 2003). Cytosolic NADH and NADPH can come from the glycolytic activity and the pentose phosphate pathway (PPP) that occurs in the cytosol and plastids (Rasmusson et al., 2020).

A possible link of the l-GalDH enzyme with the alternative electron entry may be speculated (**Figure 3**). As mentioned above, NADH can be also generated in the cytosol *via* the oxidation of l-Gal by l-GalDH (Smirnoff, 2010). This enzyme needs NAD^+^ to function and it has been suggested that the re-oxidation of NADH to NAD^+^ may limit the reaction (Gatzek et al., 2002). Indeed, the increase in l-GalDH activity does not lead to a proportional increase in the conversion of l-Gal to AsA (Gatzek et al., 2002). The hypothesis of that the NADH generated during the action of this enzyme may be re-oxidized by external NADH DHs is plausible (**Figure 3**). Intriguingly, many external NADH DHs and AsA biosynthetic enzymes are highly responsive to common stimulus such as drought and light. However, this link remains speculative considering that direct demonstration of an involvement of external NADH DHs on re-cycling the NADH derived from l-GalDH is still lacking.

## The l-GalLDH: A direct link between AsA synthesis and the mETC

There are other enzymes that can serve as electron sources but do not channel the flux *via* UQ (Millar et al., 2003). Instead, they deliver electrons directly to Cytc. Two known examples of enzymes that introduce electrons directly to mETC *via* Cytc are the l-GalLDH (Bartoli et al., 2000) (**Figure 4B**) and the proline dehydrogenase (ProDH) (Launay et al., 2019; Han et al., 2021). This Section will focus on the l-GalLDH of the AsA synthesis pathway and the possible functional implications of the link between this enzyme and the mETC.

The entry of electrons *via* Cytc by the l-GalLDH, without the direct delivery to UQ, is a crucial difference between the alternative NADH DHs and the l-GalLDH enzyme (**Figure 4B**). In addition, the l-GalLDH enzyme also differentiates from the Complex III because l-GalLDH does not seem to require UQH_2_ as carrier to transfer electrons to Cytc. By contrast, Complex III needs UQH_2_ (Millar et al., 2003). Here, we called the alternative cytochrome c reduction pathway (“ACR pathway”), the pathway of introduction of electrons through l-GalLDH (**Figure 4B**). It is to differentiate the ACR pathway from the “classical” mechanism of Cytc reduction *via* Complex III (**Figure 4A**).

The reaction of the l-GalLDH reduces Cytc but it is not the limiting step of AsA synthesis (Leferink et al., 2008; Morales et al., 2022). As mentioned above, the enzyme GDP-l-galactose phosphorylase may be apparently acting in coordination with the other biosynthetic enzymes to limit the availability of substrates (Bulley et al., 2009; Viviani et al., 2021). The total AsA amount indicates mitochondrial capacity for Cytc reduction by l-GalLDH (Alhagdow et al., 2007; Morales et al., 2014; Morales et al., 2022). However, the amount the total AsA does not reflect the rate of Cytc reduction. Analysis of plants with silenced l-GalLDH activity shows that the level of AsA does not correlate with the rate of electron flux from l-GalL to Cytc (Alhagdow et al., 2007). l-GalL or l-GulL are considered substrates for the l-GalLDH activity (Wheeler et al., 2015). Without the provision of substrates by up-stream enzymes, obviously the l-GalLDH may not transfer electrons to Cytc and consequently produce AsA. It is suggested that in mutants with low levels of AsA biosynthetic enzymes and substrate availability, the actual electron transfer through Cytc is expected to be limited.

## The mETC and the alternative Cytc reduction under the regulation of AOX pathway

With independency of the input pathway by which electrons are introduced *via* UQ, this electron carrier must be reduced. It is known that in plant mitochondria, the re-oxidation of UQH_2_ can be done by Complex III (**Figure 4A**) or by AOX pathway (**Figure 4B**). When Complex III is active, it in turn reduces Cytc, concomitantly with the proton transport into the IMS (**Figure 4A**). AOX and Complex III may be seen as competitors for the same substrate UQH_2_ (Rasmusson et al., 2020). This hypothesis implicitly considers that there is a pool of UQ and that there are not separate UQ pools for the entry of electrons through distinct sources. Thus, the more UQ pool engagement with alternative NADH DHs and AOX (**Figure 4B**), less availability of UQ for Complexes I and II (**Figure 4A**).

Unlike Complex III, the AOX pathway is by far the most regulated component of the electron transport chain. The UQH_2_ produced by the alternative NADH DHs can be preferably re-oxidized by the AOX (**Figure 4B**) whereas UQH_2_ produced by Complex I or II can be more linked with the Complex III (**Figure 4A**) (Rasmusson et al., 2020). In support of this hypothesis is that, when the AOX and alternative NADH DHs are repressed, electron flux from Complex I *via* Complex III continues (Rasmusson et al., 2020). By contrast, the chemical blocking of Complex III leads to increase of AOX, alternative NADH DHs and AsA synthesis (Bartoli et al., 2000). Dysfunctions of Complexes I, II and III often parallel with enhanced AOX (Liu et al., 2010).

Some findings also suggest a tight positive link of AsA synthesis with AOX and negative with the main electron transport pathway. For instance, increased AsA content is found in tobacco CMSII mutant with impaired Complex I (Dutilleul et al., 2003). The Arabidopsis mutant of Complex I (*ndufs4*) with low phosphorylation efficiency has elevated AsA synthesis (Meyer et al., 2009). The Arabidopsis *ppr40-1* mutant, with strongly reduced mETC through Complex III, presents higher AsA synthesis and increased l-GalLDH activity (Zsigmond et al., 2011). Plants exhibiting a down-regulation of mitochondrially localized enzymes (aconitase and malate dehydrogenase), and displaying up to 50% reduction in dark respiration, contained increased levels of total ascorbate and improved plant performance (Carrari et al., 2003; Nunes-Nesi et al., 2005; Urbanczyk-Wochniak et al., 2006). It is supposed that the alternative Cytc reduction is a prerequisite for the enhanced synthesis of AsA in these mutant plants.

These facts are consistent with the main hypothesis of this review: plant mitochondria present the “classical” pathway of Cytc reduction *via* Complex III (**Figure 4A**) competing with the ACR pathway (**Figure 4B**). At low levels of electron transport *via* the main pathway, the way for reducing Cytc and thus supporting some proton pumping and phosphorylation would be by using the ACR pathway. It is believed that the enzyme l-GalLDH may influence the respiratory process (Bartoli et al., 2000; Millar et al., 2003), bypassing the phosphorylating respiratory Complexes I and III (**Figure 4B**).

l-GalLDH activity also competes to reduce Cytc, thus a tight coordination with Complex III *via* the main respiratory transport chain may likely exist. The blocking of Complex III with a specific inhibitor, antimycin A, enhances AsA synthesis capacity (Bartoli et al., 2000). These facts are consistent with a key hypothesis of this review emphasizing the negative inter-link between the “classical” pathway of Cytc reduction (UQH_2_ to Cytc) and the ACR pathway. Both Complex III and l-GalLDH use the same substrate Cytc, thus hypothetically a high Complex III activity may be a limitation for the alternative entry of electrons through Cytc (**Figure 4B**). The decrease of electron movement from UQH_2_ to Cytc through Complex III may indirectly allow the ACR pathway to proceed and, consequently, up-regulate AsA synthesis.

The joint function of alternative NADH DHs and AOX is considered a non-phosphorylating mechanism. In relation with the activity of l-GalLDH, it has not been demonstrated if or not this enzyme leads to ATP synthesis. Complex IV is proton-pumping and there is the possibility of some proton gradient formation coupled with the alternative Cytc re-oxidation (**Figure 4B**). More ACR pathway may contribute to energy provision through some phosphorylating cytochrome c oxidase (COX) activity. Because of the bypass of major phosphorylating Complexes, it is likely a low efficiency of phosphorylation during AsA synthesis. An elevated activity of the non-phosphorylating AOX pathway is generally coupled with higher AsA synthesis (Bartoli et al., 2006). Enhanced activity of Complex IV and AOX respiratory activities were found in CMSII mutant plants with dysfunctional Complex I (Priault et al., 2007). As this co-operation has been found under light, one may suggest that the joint action of the alternative pathways of UQ and Cytc reduction may be a feature of plant response to light. Under light conditions, the changes in the main respiratory pathway and Krebs cycle and the possible decline in ATP synthesis may be, in part, compensated with an increase of AsA synthesis. Otherwise, if the alternative NAD(P)H dehydrogenases together with AOX (non-phosphorylating pathways) are active under light but lack the ACR pathway, ATP synthesis would not be formed. Limited alternative Cytc reduction and possible low energy provision would be the case occurring in plants with silenced l-GalLDH and high AOX capacity, which presented growth defects (Alhagdow et al., 2007). Interestingly, respiratory mutants of Complex I such as *ndufs4* and CMSII mutants have lower ATP in dark (as expected due to the dysfunctional Complex I), however, they show higher ATP level and AsA accumulation under conditions with light (Szal et al., 2008; Meyer et al., 2009). Unfortunately, the ATP production capacity in low AsA mutants has not yet been examined.

The hypothesis of a tight co-operation between the alternative pathways is also supported by further correlative findings. Studies have showed that lower AOX contribution increases the energy efficiency of respiration under light-limiting conditions (Noguchi et al., 2001). Increased carbon-use efficiency was observed under phosphorus/nitrogen stress in AOX-suppressed cells of *N. tabacum* (Sieger et al., 2005). Limitations of light and phosphorus are conditions that affect AsA synthesis (Zhang et al., 2020; Maruta, 2022). It is speculated that low AOX may improve plant performance under conditions where the ACR pathway is expected to decline.

## Cytochrome c and COX may not be main limitations for AsA synthesis

As mentioned above, the enzyme GDP-l-galactose phosphorylase is considered the limiting factor for AsA synthesis. It was here suggested that the low availability of precursors of AsA (particularly, l-GalL) may limit the l-GalLDH activity. On the other hand, the enzyme COX catalyzes the reaction by which electrons in the Cytc are transferred to oxygen. As suggested above, Complex III may be a limitation for alternative Cytc reduction. A low amount of Cytc and COX activity may also limit the rate of alternative Cytc reduction. The absolute need of Cytc for AsA synthesis is now under debate. When redox status of mETC is high, i.e., when Cytc is completely reduced in active mitochondria, the AsA synthesis by the enzyme is not observed (Millar et al., 2003). However, it has been seen that in *CYTC* mutants of *A. thaliana* lowing Cytc in mETC, AsA accumulation is not affected. Even an increase of approximately 3-fold in AsA accumulation in response to the high light cycles is found, although there is a 60% reduction in l-GalLDH activity of these mutant plants (Welchen et al., 2012). Furthermore, in fruit mitochondria, the block of Complex IV (cyanide-sensitive respiration) maintains significant AsA synthesis capacity (Morales et al., 2022), further suggesting that AsA synthesis may function with lower Cytc availability. The discrepancy between the Millar’s experiments and the others may be explained due to AOX capacity. All experiments in which Cytc was not a limitation for AsA synthesis showed higher AOX capacity. According to our model, one possible explanation for enhanced AsA synthesis is that the effect of Complex III limitation for alternative Cytc reduction is weak when AOX is present.

A new perspective about the functioning of mETC may be under scrutiny in the future. The most accepted view is that, in parallel with Complex IV activity, AOX activity is able to correct metabolic imbalances that occur during phosphorylation at high availability of reduced equivalents or excessive ATP production (Vanlerberghe et al., 2020). However, we added to this predominating view the fact of that plant mitochondria alternatively receive electrons directly from AsA synthesis *via* Cytc. Unexpectedly, it was demonstrated that the excessive rate of alternative Cytc reduction can alter the “typical” respiratory pattern of plant mitochondria (Morales et al., 2022). Imbalance can be caused by this alternative source of electrons *via* l-GalLDH. The role of AOX may also imply the protection against imbalanced ACR pathway. Interestingly, several respiratory mutants such as CMSII that enhance AsA level lack the typical electron partition to AOX (Vidal et al., 2007). It is plausible that the proposed ACR pathway can help explain why the control respiratory does not play a significant role in plants or why the over-expression of AOX can lead to increase of COX under specific stress conditions (Dahal and Vanlerberghe, 2017). AOX can act to maintain the COX function (Dahal and Vanlerberghe, 2017) and this effect may occur with the participation of l-GalLDH activity.

## The electron transport and the generation of ROS associated with the alternative Cytc reduction

It is known that some ROS is unavoidably produced when an unbalanced electron flux takes place. The mitochondria represent the most active ROS-generating center in heterotrophic plant cells; between 2 to 5% of all O_2_ consumed by the organelle is used for the production of ROS (Gupta et al., 2015). The mETC activity is responsible for ROS generation (i.e. H_2_O_2_ and O_2_^•-^), mainly through Complexes I (NADH-ubiquinone oxidoreductase) and III (ubiquinone: cytochrome c oxidoreductase) (Møller, 2001; Sweetlove and Foyer, 2004; Gupta and Igamberdiev, 2015). In isolated plant mitochondria with available ADP and in the presence of uncouplers, H_2_O_2_ accumulation correlates negatively with electron transport rates (ETR) and positively with membrane potential (Purvis, 1997; Møller, 2001).

The O_2_^•-^ generated by unbalanced mETC activity or spontaneously from O_2_ with the participation of Fe-S proteins, can also be dismutated into H_2_O_2_ by the enzyme mitochondrial superoxide dismutase (Mn-SOD) (Gill and Tuteja, 2010). The generated H_2_O_2_ can be dismutated into H_2_O and O_2_ through catalase, present mainly in the peroxisome and possibly also in the mitochondria (Scandalios et al., 1980; Heazlewood et al., 2004; Mhamdi et al., 2010). H_2_O_2_ can cross membranes by its interaction with aquaporins (Bienert et al., 2007). H_2_O_2_ and O_2_^•-^ interact and lead to the generation of other ROS such as hydroxyl radical, OH^•^, more deleterious to cell metabolism. This is one of the most reactive ROS, which can oxidize nucleic acids and proteins and lead to lipid peroxidation (Rigo et al., 1977; Foyer et al., 1997; Sharma et al., 2012). OH^•^ has a half-life of 10^-9^ s and it is quite reactive.

The likelihood of an oxidative stress enhances if unbalanced electron fluxes occur. An uncoupling between the electron transfer to Cytc *via* l-GalLDH and the mETC may potentially lead to electron leakage to oxygen. The excess of electron flux through the ACR pathway may be a ROS generator in plant mitochondria (Han et al., 2021; Morales et al., 2022). Substrates such as l-GalL and proline induce the mitochondrial ROS formation. Indeed, a recent work demonstrates that an over-reduction of Cytc by excessive l-GalLDH activity can generate ROS (Morales et al., 2022).

The mechanism of electron transfer by l-GalLDH may explain the observation of ROS formation during an increase of the rate of alternative Cytc reduction. l-GalLDH is a FAD sugar oxidoreductase or aldonolactone oxidoreductase enzyme from the vanyl-alcohol oxidase (VAO) family of flavoproteins (Leferink et al., 2008). It is believed that l-GalLDH does not generate H_2_O_2_ during its action (Smirnoff, 2018). However, because the mechanism of electron transfer may imply two-electron to one-electron transfer (between FAD and Cytc), theoretically ROS might be generated. ROS may affect the enzyme because the l-GalLDH requires redox-sensitive thiol for optimal AsA synthesis (Leferink et al., 2009). A proposed role of AOX is to act as an antioxidant, reducing the possibility of ROS production. AOX receives electrons directly from UQH_2_, transferring them to O_2_ without passing through Complexes III and IV. Electrons are transferred through UQH_2_ to the partial O_2_ reduction that results in O_2_^•-^ generation by Complexes I and III, due to the interaction of these Complexes with UQH_2_ (Goncalves et al., 2015; Patterson et al., 2015). Thus, the greater the UQH_2_ pool, the greater is ROS production, which can be even higher when Complex III is inhibited (Goncalves et al., 2015). By limiting the electron entry to Cytc from UQH_2_ through Complex III, the AOX can decline the likely ROS preventing Cytc over-reduction.

AOX uncouples UQH_2_ reoxidation from cytochrome reduction (Vanlerberghe et al., 2016; Del-Saz et al., 2018). Thus, AOX strongly controls the production of ROS in the cellular environment (Maxwell et al., 1999; Amirsadeghi et al., 2006; Yoshida et al., 2007; Vanlerberghe, 2013; Garmash, 2021). It was demonstrated that the *in vitro* ROS level induced by the AsA substrate, l-GalL, declines if AOX is activated. On the other hand, when AOX is inhibited by SHAM, both l-GalLDH activity and AsA production are negatively affected (Morales et al., 2022). According to the hypothesis we arise in this review, when AOX is active, the main electron flow *via* complex III declines, keeping Cytc oxidized and available to receive electrons through the alternative pathway from l-GalLDH (**Figure 4B**). AOX has been associated with enhance of l-GalLDH activity under light conditions (Bartoli et al., 2006). However, when AOX is inactive, l-GalLDH activity may generate ROS in view of the greater probability of excessive reduction of Cytc by Complex III. Unfortunately, there are no data evaluating the effect of *in vivo* suppression of AOX on l-GalLDH activity. Thus, the view is that in plant mitochondria the redox state of mETC also depends on the rate of alternative electron entry during AsA synthesis.

Some mutants of mETC with low levels or dysfunctional Complexes I and III often show elevated AsA synthesis and concomitantly enhanced ROS production and higher AOX expression (Meyer et al., 2009). The stress severity in these mutants largely depends on light. A reduced light period alleviates stress and long light exposure accelerates stress (Liu et al., 2010). In general, they have greater ROS level and higher AOX expression, which correlates with higher tolerance to different stress for cases in that it was measured (Liu et al., 2010; He et al., 2012; Yang et al., 2014). Greater ROS production was observed into the mitochondrial intermembrane space of the mosaic MSC16 mutant, which presents a dysfunctional Complex I and enhanced AsA level (Szal et al., 2009). Both some mutants with low AsA and also respiratory mutants have signs of ROS over-production. Besides, both types of mutant plants present clear growth defects. However, the difference is that many respiratory mutants with higher AsA level and enhanced ROS show tolerance to abiotic stress whereas the low AsA mutants are stress- sensitive. If or not AOX is involved in defining the difference in stress sensitivity has not been yet examined. AOX1 can be induced by various stresses and is a marker for mitochondrial retrograde response (Giraud et al., 2009). Likely, the induction of ROS during imbalanced AsA synthesis can lead to such response of AOX.

Morales et al. (2022) also observe that *in vitro* mitochondrial ROS production can occur in parallel with significant AsA synthesis and altered mETC. The supposition of that an imbalanced ACR pathway concomitantly with the AsA synthesis leads to ROS over-production is consistent with the hypothesis of that the rate of electron entry during AsA synthesis is likely an important determinant of mitochondrial redox state overall and the amount of ROS produced. The production of ROS is higher under stress conditions (Møller, 2001; Blokhina and Fagerstedt, 2010), which should demand greater antioxidant activity of AsA (Foyer and Noctor, 2011; Rosado-Souza et al., 2020). However, the higher AsA amount in the respiratory mutants was not able to efficiently overcome the endogenous ROS stress observed (Meyer et al., 2009). The ACR pathway would be co-operating with the activity of Complex IV, but at the expense of lower electron flux *via* Complex III, which would lead to an increase in the UQH_2_ pool. This means that if there is a very intense stimulus to AsA synthesis, the respiratory process *via* Complex III can be negatively influenced, i.e., a lower oxidative phosphorylation, which could even increase the production of ROS if enough AOX is not ensured (Wheeler et al., 1998; Bartoli et al., 2000; Millar et al., 2003; Morales et al., 2022). Under normal conditions this possibility is quite remote, but under stressful conditions that stimulate l-GalLDH such as high light, it is likely (Bartoli et al., 2000; Morales et al., 2022).

## Defects of biosynthetic mutants with low AsA: Possible links with mETC

The production of defective mutants for enzymes of this pathway has shown limiting steps in the AsA synthesis pathway (Radzio et al., 2003; Conklin et al., 2006; Linster et al., 2007). Mutants such as *vtc1* (defective in GDP-mannose phosphorylase), *vtc2* and *vtc5* (defective in GDP-l-galactose phosphorylase), and *vtc4* (defective in l-galactose-phosphate phosphatase), accumulate about 20 to 80% of the accumulated AsA by wild plants. Until now, no viable plant that totally lacks AsA has been identified.

All these mutants show multiple defects and have their metabolism compromised, especially when exposed to stressful conditions, such as exposure to intense light or ozone (Veljovic-Jovanovic et al., 2001; Müller-Moulé et al., 2003; Müller-Moulé et al., 2004; Dowdle et al., 2008). As AsA is the antioxidant more abundant in plant tissue, authors have suggested that such defects may be primarily caused by a low antioxidant capacity and altered redox regulation in plants with low AsA content (Kiddle et al., 2003). However, it is now recognized that other metabolic and signal pathways may also mostly contribute to the phenotype of *vtc* mutants (Kerchev et al., 2011). Some features of these mutants do not seem to be fully specific of a low AsA level so there are defects cannot be explained by the AsA deficiency per se (Barth et al., 2010). In addition, although studies have shown that the phenotype of low AsA plants can be rescued by the supplementation of AsA precursors, some responses to AsA or l-GalL precursor are quite different in terms of gene expression (Bulley et al., 2021) and when examined using *in vitro* mitochondria (Morales et al., 2022). Furthermore, antioxidant compensatory mechanisms under AsA deficit (example, enhanced glutathione content) can be expressed in these mutants (Smirnoff et al., 2000; Pavet et al., 2005), which can mask the AsA-specific response.

However, in this section of the review, we will attempt to connect some defects observed in low AsA mutants with possible changes in the respiratory pathways. According to the proposed hypothesis, it becomes clear that the alterations of low AsA mutants may also be explained by changes in the respiratory pathways.

The first two mutants, *vtc1* (Conklin et al., 1996) and *vtc2* (Jander et al., 2002), were isolated because of their sensitivity to ozone. This effect was initially attributed to a lack of antioxidant protection in tissues with low AsA. However, ozone sensitivity may be also result of imbalances in the respiratory pathways and ROS production under a low AsA synthesis background. It is known that a profound decrease of COX pathway is accompanied by an increase in AOX activity.

This same rationality can be applied to the analysis of other stresses. For example, salt and drought decline AsA content (Smirnoff et al., 2000). Increased sensitivity to salinity/drought and higher stress-induced ROS production was also found in low AsA mutants (Huang et al., 2005; Niu et al., 2013). Salt and drought enhance AOX pathway (Clifton et al., 2006). Based on the hypothesis, it may be speculated that a low AsA during stress in cooperation with enhanced AOX generate ROS. As was suggested above, an increase of AOX under a context of low AsA synthesis may be negative for plant performance.

By contrast, high light stress in plants can induce AsA synthesis and the effect of this type of stress may be quite distinct. Unlike ozone or salt stress, light enhances both AsA synthesis and AOX1 (Strodtkötter et al., 2009; Garmash et al., 2020). It is well-known that high light up-regulates several genes related to AsA synthesis (Tabata et al., 2002). According to the proposed model, the occurrence of alternative Cytc reduction together with the increase of AOX may become plants with better capacity to acclimate to light conditions. As cited above, the co-regulation of AsA and AOX under light conditions contribute to a better growth performance (Bartoli et al., 2006).

At present, it can be only speculated that AOX may have different effects under conditions linked with low or high AsA synthesis. A published study shows that higher AOX capacity and stress signs are present in the tomato plant with silenced l-GalLDH enzyme (Alhagdow et al., 2007). However, to the best of our knowledge, there are not works showing *in vivo* evidence of the activity of AOX pathway in low AsA mutants.

## Final considerations and future prospects

In this review, we seek to open a new perspective to focus the known link between the mitochondrial function and AsA synthesis. The tight relationship between the AsA synthesis and the mitochondrial electron transport chain has been previously proposed (Bartoli et al., 2000; Millar et al., 2003). However, we stressed the potential implications of this this link for plant mitochondria. This is an issue that has received relatively little attention in previous reviews. The last step of the AsA synthesis, catalyzed by the l-GalLDH enzyme (Bartoli et al., 2006), can be considered as an extra-mitochondrial source of electrons for the mETC. We argument that the provision of electrons *via* l-GalLDH is unique in terms of that the electron supply is introduced directly to the Cytc. This feature of plant mitochondria is likely to have an impact in plant respiration as evidenced by phenotypes showing both altered respiratory activity and AsA synthesis capacity. Also, alterations of AsA synthesis with specific mitochondrial inhibitors have allowed to suggest possible connections of mitochondria with AsA synthesis (Bartoli et al., 2006; Morales et al., 2022).

Despite plants with low AsA have been characterized in the last years (Veljovic-Jovanovic et al., 2001; Müller-Moulé et al., 2003; Müller-Moulé et al., 2004; Dowdle et al., 2008), a few studies have focused in-depth analysis of mitochondrial functions in these mutants. We considered that future works should attempt to obtain a better understanding about mitochondria function in the context of low AsA synthesis. The observed phenotypes of low AsA mutants may likely be result of long-lasting effects of mutations on the respiratory activity, particularly the alteration of the alternative pathways. Phenotypes such as sensitivity to ozone, salinity, high light, photo-inhibition, accelerated senescence and others may reflect compensatory mechanisms of the mitochondrial respiration in response to endogenous low AsA synthesis rather than the lack of AsA-antioxidant capacity. In addition, double mutants with defects in genes related to AsA synthesis, transcriptional factors, hormone synthesis and signaling, and ROS signaling may be needed to dissect the specific regulatory cross-talks between the components of the respiratory pathways and plant signaling during AsA synthesis.

Finally, it is hoped that this review can inspire new discussions and open new research avenues that relate AsA synthesis with its functions in plant metabolism. The genetic and chemical manipulation of the mitochondrial activity may be a useful tool to improve the AsA synthesis and eventually the tolerance of plants to abiotic and biotic stress. Modifying AsA synthesis and respiratory activity might be a strategy for the conservation of plant products, particularly in the case of products of commercial interest (Dusenge et al., 2018; Collalti et al., 2019; Jalali et al., 2020). In conclusion, the involvement of AsA in regulating multiple plant functions goes beyond simply its roles as antioxidant and co-factor molecule.

## Author contributions

LMMM and JGO proposed the ideas, planned, wrote and revised the manuscript. IFM, DBS, GMCS, MMAG, RAA, and JHC reviewed and JGO edited the manuscript. All authors contributed to the article and approved the submitted version.

## Conflict of interest

The authors declare that the research was conducted in the absence of any commercial or financial relationships that could be construed as a potential conflict of interest.

## Publisher’s note

All claims expressed in this article are solely those of the authors and do not necessarily represent those of their affiliated organizations, or those of the publisher, the editors and the reviewers. Any product that may be evaluated in this article, or claim that may be made by its manufacturer, is not guaranteed or endorsed by the publisher.
